# Spatiotemporal Patterns of Anthrax, Vietnam, 1990–2015

**DOI:** 10.3201/eid2811.212584

**Published:** 2022-11

**Authors:** Morgan A. Walker, Luong Minh Tan, Le Hai Dang, Pham Van Khang, Hoang Thi Thu Ha, Tran Thi Mai Hung, Ho Hoang Dung, Dang Duc Anh, Tran Nhu Duong, Ted Hadfield, Pham Quang Thai, Jason K. Blackburn

**Affiliations:** University of Florida, Gainesville, Florida, USA (M.A. Walker, L.M. Tan, T. Hadfield, J.K. Blackburn);; National Institute of Hygiene and Epidemiology, Hanoi, Vietnam (LH. Dang, P.V. Khang, H.T.T. Ha, T.T.M. Hung, H.H. Dung, D. Duc Anh, T.N. Duong, P.Q. Thai)

**Keywords:** anthrax, Bacillus anthracis, bacteria, spatiotemporal patterns, epidemiology, incidence, mortality rate, vaccine-preventable diseases, zoonoses, Vietnam

## Abstract

Anthrax is a priority zoonosis for control in Vietnam. The geographic distribution of anthrax remains to be defined, challenging our ability to target areas for control. We analyzed human anthrax cases in Vietnam to obtain anthrax incidence at the national and provincial level. Nationally, the trendline for cases remained at ≈61 cases/year throughout the 26 years of available data, indicating control efforts are not effectively reducing disease burden over time. Most anthrax cases occurred in the Northern Midlands and Mountainous regions, and the provinces of Lai Chau, Dien Bien, Lao Cai, Ha Giang, Cao Bang, and Son La experienced some of the highest incidence rates. Based on spatial Bayes smoothed maps, every region of Vietnam experienced human anthrax cases during the study period. Clarifying the distribution of anthrax in Vietnam will enable us to better identify risk areas for improved surveillance, rapid clinical care, and livestock vaccination campaigns.

Pathogens that persist in environmental reservoirs represent a major and underappreciated risk for humans and animals (*1*). *Bacillus anthracis*, the causative agent of anthrax, is an extreme example of environmental pathogen persistence because its spores persist for long periods (*2*), and indirect transmission from environment-to-host is obligate (*3*). Outbreaks are documented nearly worldwide, and the distribution of disease is constrained by specific environmental conditions (e.g., soil pH, organic matter, calcium) (*2*,*4*,*5*). Outbreaks generally arise in steppe/grassland habitats in wildlife populations (*6*) and livestock; this pattern was modeled globally (*7*), nationally (*8*–*13*) and locally (*14*–*16*) for several regions. The primary hypothesized infection route for livestock/wildlife is ingestion of *B. anthracis* spores during feeding at sites in which spores are concentrated (*17*). Human cases are primarily results of spillover from animal cases, particularly by handling carcasses or meat of livestock (*18*) or wildlife (*19*,*20*). Anthrax remains a major disease in developing countries in Africa and Asia (*21*,*22*). Where present, anthrax is major factor in public health (*23*), food web dynamics (*24*), and wildlife conservation (*25*).

An estimated 20,000‒100,000 human cases of anthrax occur annually worldwide, mostly in poor rural areas (*26*). Cutaneous exposure to *B. anthracis* accounts for most human cases worldwide, typically with low mortality rates; gastrointestinal exposure shows intermediate-to-high case-fatality rates. Cutaneous and gastrointestinal cases of anthrax are most commonly caused by handling and slaughtering infected livestock or butchering and eating contaminated meat; untreated gastrointestinal cases account for most human deaths (*4*,*21*).

In Vietnam, anthrax has been identified as a priority zoonotic disease for control in a joint Ministry of Health and Ministry of Agriculture and Rural Development Circular (#16, 2013) (http://vbpl.yte.gov.vn/van-ban-phap-luat/TTLT-162013ttlt-byt-bnnptnt-.12.1706.html#pdf). Disease reports of anthrax in Vietnam in the literature date to the 1940s, with reports of agricultural risk for terrace-working farmers (*27*) (a dominant farming practice across much of current-day, mountainous rural Vietnam). Historically, anthrax foci were defined in southern Vietnam and along the northern border with China. Today, anthrax appears concentrated in 6 northern provinces, 5 of which border China (the Northern Midlands and Mountainous region) (Figure 1), with few reports from southern Vietnam (*28*). Several recent studies in China have reported sustained, as well as increasing areas of moderate human and livestock anthrax in provinces bordering northern Vietnam (*29**,**30*), an area with known transborder trade and livestock markets (temporarily restricted because of the COVID-19 pandemic). Generally, surveillance is anthropocentric with limited livestock reporting; comparable records are not currently available for livestock. Therefore, anthrax burden is unknown/underestimated, and the geographic distribution of anthrax in Vietnam remains to be defined, challenging our ability to identify target areas for control.

**Figure 1:**
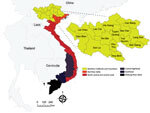
Regions of Vietnam. Current reports of anthrax in Vietnam are concentrated in the Northern Midlands and Mountainous region (inset), especially in 6 provinces bordering or close to China: Dien Bien, Lai Chau, Lao Cai, Ha Giang, Son La, and Cao Bang.

Because most human infections with anthrax are caused by contact with infected animals or their byproducts (e.g., meat or hides), targeting livestock with annual vaccination is the most effective method to control anthrax in animals and, consequently, in humans (*31**‒**33*). Despite the effectiveness of vaccination, anthrax persists in areas with weakened health infrastructures; as a result, long-term vaccination strategies are often needed in disease-endemic areas (*31*). To prioritize areas for vaccination campaigns and disease surveillance and control, an understanding of risk areas is a necessity. To clarify anthrax risk areas in Vietnam, we retrospectively analyzed human anthrax case data for 1990‒2015. We calculated nationwide and province-level anthrax incidence rates for this period, with the goal of assessing disease burden, a first step to prioritizing risk areas for management.

## Methods

### Epidemiologic Data

We extracted province-level data on human anthrax cases for 1990–2015 from the Vietnam Health Statistics Yearbooks published for 1991‒2016 by epidemiologists of the National Institute of Hygiene and Epidemiology, Ministry of Health, Vietnam. Before 2015, anthrax was reported on weekly, monthly, and annual bases from commune health centers and district hospitals to District Medical Centers. From there, weekly, monthly, and annual reports were provided to the Provincial Preventative Medicine Centers, which reorganized into the Provincial Centers for Disease Control as of 2015. Reports of Provincial Preventative Medicine Centers/Provincial Centers for Disease Control were submitted monthly and annually to National Institute of Hygiene and Epidemiology and 3 other regional institutes corresponding to each region of Vietnam. The institutes reviewed, compiled, and submitted the annual data to the Ministry of Health for further compilation and publication together with other health data. Anthrax was made reportable within 24 hours in 2015 as a class B infectious disease by circular number 54/2015/TT-BYT issued by the Ministry of Health (*34*).

### Population Data

We obtained population data for the provinces of Vietnam for 2000‒2015 from the WorldPop population counts database (*35*). This database incorporates census and open access ancillary data in a random forest estimation technique. The random forest model generates a gridded prediction of population density at 100-meter spatial resolution, which is used as a weighting surface to perform dasymetric redistribution, resulting in pixel-level census counts available for the whole country (*35*).

We aggregated these gridded population data to the provincial level by using the zonal statistic routine in QGIS 3.8 (https://www.qgis.org). In this instance, the provinces of Vietnam acted as the polygon layer, and the pixels of population data in each province were summed by using the zonal statistic to achieve a final calculation of the population of each province. The population was calculated by using this method for each province during 2000‒2015. However, because WorldPop data are not available for years before 2000, we used a different approach for 1990‒1999. For these years, we back calculated the population by using the United Nations average annual rate of change (*36*) for 2000‒2001 and applying it to the provinces (Appendix Equation).

To verify the accuracy of this approach, we compared census population data collected by the country of Vietnam with the WorldPop population dataset. Census data from the 2019, 2009, and 1999 censuses were publicly available. We provide a comparison of population data from the 2 datasets, as well as the national incidence of anthrax cases (Appendix Figure 1). The WorldPop estimate is slightly higher than the census population data, especially for 1995‒2005. However, our national incidence rate calculations were nearly identical regardless of the population estimate used (Appendix Figure 1).

The administrative boundaries of the provinces of Vietnam have changed several times since 1980 (*37*,*38*). During our study period, splits or merges occurred in 1990, 1991, 1992, 1997, 2004, and 2008 (Appendix Figure 2). During 1990‒2015, the number of provinces in Vietnam increased from 44 to 63 (*39*). These administrative boundary changes were considered when calculating the populations of each province as outlined; thus, the zonal statistic was used on different polygon layers that corresponded to the provincial boundaries of that year. Administrative boundaries of the choropleth maps in the results are also displayed accurately to the corresponding year.

Once population data were available as denominators, we plotted total cases and incidence per 10,000 persons annually for all of Vietnam. We also fitted a linear trend for each case and incidence in Excel 365 (Microsoft, https://www.microsoft.com).

### Spatial Incidence Mapping

For mapping, we calculated provincial level human anthrax incidence rates annually for 1990‒2015. We obtained incidence rates by dividing raw cases numbers in each province by the population of each province and multiplying by 10,000 for each year. Accordingly, all incidence rates reported are per 10,000 persons. We spatially smoothed raw incidence rates to improve estimates of anthrax cases that might have gone unreported.

Smoothing is a method of statistically adjusting the estimate for the underlying risk in each spatial unit by using information provided by the other spatial units (*39**,**40*). When subdividing national estimates into individual provinces, variance estimates can be unstable (*41*), and instability is increased in rural areas. The goal of smoothing is to adjust rate estimates toward a global or local mean, with a larger effect on spatial units (here, provinces) that have smaller populations (*39*). We applied spatial Bayes in GeoDa 1.20 (*39*). In brief, spatial Bayes smoothing uses the raw rate for each areal unit averaged with a localized reference estimate, the extent of which is based on a weights matrix. We used a first-order queen contiguity weights matrix, which defines the neighbors of a location as those that have either a shared border or vertex with the polygon of interest (*39*).

We compared empirical Bayes smoothing, which adjusts values to the global mean (all of Vietnam) to spatial Bayes, which adjusts to the local mean defined by the weights matrix, reducing the adjustment to the mean incidence of immediate neighbors. For incidence rate smoothing comparisons, we chose the years with the lowest (1990) and highest (2011) incidence rates, as well as 4 additional, randomly chosen years (Appendix Figure 3). Box plots showed that spatial Bayes and empirical Bayes smoothing were similar, but spatial Bayes outperformed empirical Bayes in collapsing lower percentile outliers, the SD, and the mean (Appendix Figure 3). Accordingly, spatial Bayes smoothing was chosen for use in this study.

After smoothing, we constructed choropleth maps of the province-level incidence rates by using ArcGIS Pro 2.4.0 (https://support.esri.com). To evaluate results, we mapped each year separately (Appendix Figure 4) and developed an animated GIF enabling us to view interannual variability (Appendix Figure 5). We mapped a selection of years to illustrate areas of sustained anthrax and the wider geography of reported human anthrax over the 26 years.

## Results

### National Incidence of Human Anthrax

**Figure 2:**
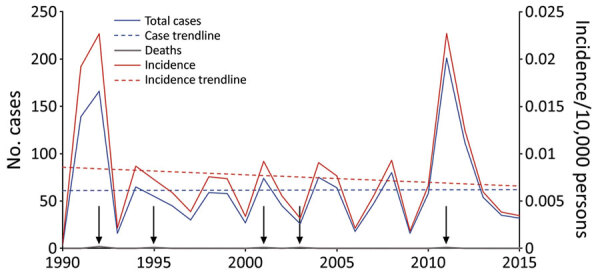
National human anthrax cases and incidence per 10,000 persons per year in Vietnam, 1990‒2015. Gray arrows indicate deaths.

**Figure 3:**
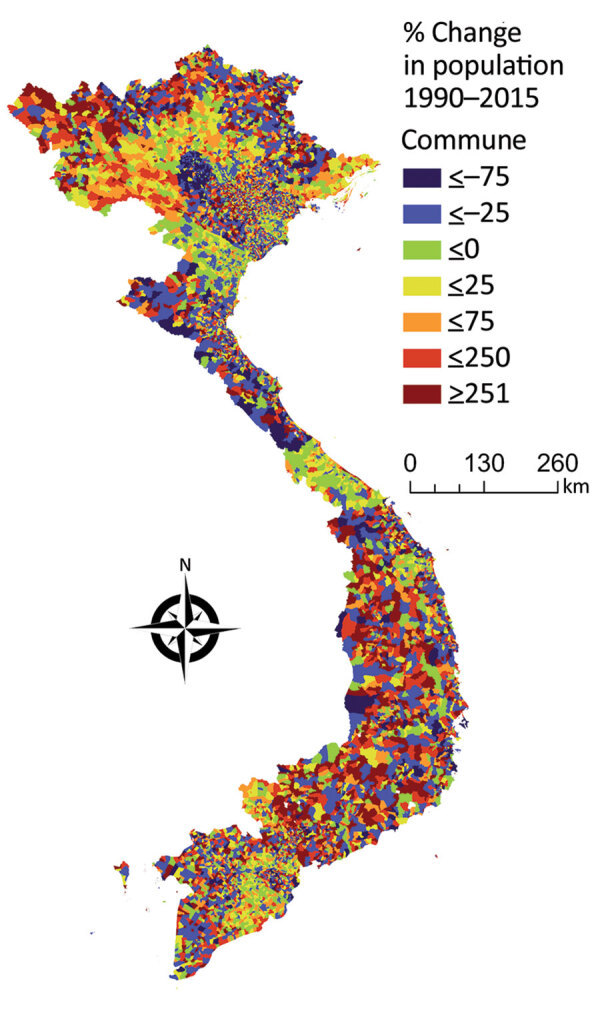
Percentage population change in the communes of Vietnam during 1990‒2015. Light blue, dark blue, and green values indicate communes that had population decreases, and yellow, orange, and red values indicate communes that had population growth.

During 1990‒2015, Vietnam reported 1,600 human anthrax cases with an annual average of 61.5 cases (Figure 2). During the study period, human deaths were reported in 1992, 1995, 2001, 2003, and 2011. Some years had >200 cases, and deaths were not necessarily in severe years (Figure 2). The trendline for cases remained at ≈61 cases per year throughout the 26 years of the study period. The trendline for incidence showed a slight decrease over time, probably a reflection of the increasing population in Vietnam (Figure 3). Years with the highest number of human cases were 1992 (166 cases) and 2011 (201 cases), reflecting large outbreaks early and late in the study period. In 1992 and 2011, the incidence rate reached 2.3 cases/10,000 persons. Between these 2 large outbreak years, incidence fluctuated with peaks every 3‒to 4 years.

### Provincial Incidence of Human Anthrax

**Figure 4:**
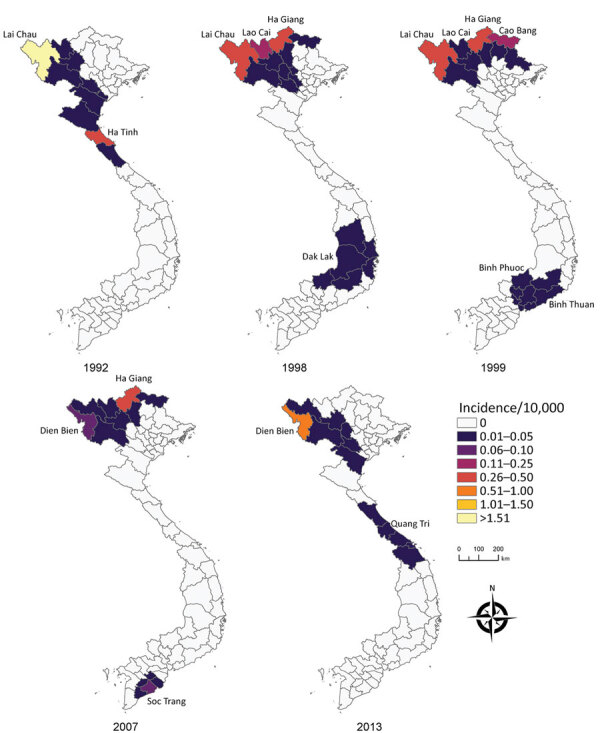
Choropleth maps of spatial Bayes smoothed human anthrax incidence rates in provinces of Vietnam. The years are not necessarily those with the highest anthrax incidence rates but those with the most widespread range of anthrax. Although anthrax incidence rates were highest in the northern provinces, they were not limited to those provinces.

Of the 63 total provinces in Vietnam, 20 provinces reported >1 human anthrax case during 1990‒2015. Four provinces reported >1 death. Most cases were reported in the Northern Midlands and Mountainous region (Figure 1), but smoothed maps identified case incidence in several years in the Red River Delta, North Central and Central Coast, Central Highlands, South East, and Mekong River Delta regions (Figure 4). The provinces of Lai Chau, Dien Bien, Lao Cai, Ha Giang, and Cao Bang had some of the highest incidence rates. Dien Bien had the highest incidence rate of all provinces in 2011 (2.62 cases/10,000 persons). Of the North Central and Central Coast region of Vietnam, Ha Tinh was the province with the highest incidence rate (0.33 cases/10,000 persons in 1992). In the Central Highlands region, Dak Lak had the highest incidence and in the South East region Dong Nai was the province that had the highest incidence. Anthrax incidence was highest in, but not exclusive to, the northern provinces (Figure 4). Anthrax incidence was widespread throughout the country during our study (Appendix Figure 4).

## Discussion

We examined the interannual patterns of human anthrax in Vietnam at the national and provincial level for 1990‒2015. There was no annual decrease in reported human anthrax cases nationally over the 26 years for which data were available. Although the national incidence rate decreased slightly during 1990‒2015, this decrease was probably caused by an increase in the population of Vietnam, rather than a decrease in raw case numbers (Figure 3). For example, the median percentage change of the population in the communes of Vietnam during 1990‒2015 was 11%. Furthermore, 56% of communes had a population increase, and 1,200 communes had a population increase of >500%. The increasing population and steady case numbers indicate that over our study period, control efforts did not effectively reduce disease burden nationally. In addition, the years in which deaths occurred (Figure 2) did not necessarily correspond to the years with the highest incidence rates, suggesting that deaths are driven by access to healthcare or knowledge of disease, rather disease intensity.

Historically, anthrax foci have appeared concentrated at the northern border with China, in the Northern Midlands and Mountainous region of Vietnam. Our study supports this finding because some of the highest incidence rates were found in the provinces of Lai Chau, Dien Bien, Ha Giang, Cao Bang, Lao Cai, and Son La. Of these provinces, only Son La does not have a border with China. However, Son La and Dien Bien both have a border with Laos. Borders that serve as areas of international transit and trade might play a major role when addressing disease control. Because *B. anthracis* is most commonly transmitted to humans through infected livestock, trading animals or meat across borders could be a cause for concern. Although this practice has been limited by COVID-19 restrictions since 2020, transnational livestock trade is a major industry in Vietnam (*42*). For example, Turner (*42*) reported how regular trade in buffalo, which are vital farming tools for ploughing terraced ﬁelds, spans the China‒Vietnam border and takes place through legal and illegal routes. On legal paths, buffalo are inspected at border checkpoints for disease, but other traders use secret routes to smuggle buffalo without permits (*42*). Livestock trade also occurs at the Laos‒Vietnam border because Lao is an importer and exporter of cattle and buffalo and a transit country for livestock destined for Vietnam and China (*43*).

Recent disease reporting from China has shown high incidence of human cutaneous anthrax in southwestern China, including Yunnan and Guangxi Provinces, which border northern Vietnam (*29**,**30*). In contrast, although anthrax is a reportable disease in Laos, publicly available data on human anthrax cases are limited (*44**,**45*). Of the provinces in Laos that reported outbreaks during 1984‒2010, none of them border the northern provinces of Vietnam where high incidence rates were reported from our study (*45*). However, this finding could be a case of underreporting and data inaccessibility, rather than an indication that anthrax outbreaks have not occurred in northeastern Laos.

Although most reported anthrax cases and the highest anthrax incidence were found in the Northern Midlands and Mountainous regions of Vietnam, our study shows that human anthrax incidence is much more widespread throughout the country; smoothed rate maps showed that all regions of Vietnam have probably had anthrax cases during the study period (Figure 4). This major finding helps identify risk areas and target regions for public health intervention. Furthermore, because of the ability of *B. anthracis* to form long-lasting spores resistant to multiple environmental conditions (*46*), cases occurring in these other regions of Vietnam are a good indication of the presence of *B. anthracis* in the environment. Therefore, cases could reoccur in these areas, even if outbreaks have not been reported in recent years. In addition, because of limited data available on the domestic livestock trade within Vietnam, it is unknown how movement of livestock within the country contributes to anthrax incidence. Domestic trade and transportation of draft and livestock animals from regions with a high burden of disease could contribute to the sporadic occurrence of anthrax cases in other regions.

As for all neglected zoonoses, our data probably represent an underestimation of true disease burden, which is a limitation of our study. Although anthrax is a reportable disease in Vietnam (*34*), it might go unreported because of a multitude of reasons, including lack of public awareness, stigma, or travel distance to a health provider. Case identification is also dependent on the diagnostic capacity existing in the clinical and laboratory chain down to the local level. A breakdown in any of these steps might result in underreporting of anthrax cases.

Previous research has shown that human anthrax rates increase with limited vaccination of livestock (*47*) and a decrease in sustained livestock vaccination (*48*). Although there is national policy on livestock vaccination for Vietnam, it is not clear how vaccination rules and distribution of livestock vaccines differ between provinces. Goletti et al. (*49*) found that although the supply of vaccines is not a constraint within the country, their price and quality might impede their effective use. Furthermore, limited animal health knowledge at the farm and field service levels is a key factor in the low adoption of proven disease control measures. More data on the distribution and use of anthrax vaccines is needed in Vietnam and worldwide (*7*).

In conclusion, the current anthrax situation in Vietnam remains a public and veterinary health threat because of challenges with reporting, surveillance, and control. Our findings suggest anthrax has occurred throughout Vietnam, and the highest incidence are in provinces of the Northern Midlands and Mountainous region. Future control efforts need to focus on improving (and reporting) livestock vaccination rates, as well as advancing public awareness and knowledge of the disease, especially in these risk areas. The interconnectedness of humans, livestock, and wildlife is evident when examining anthrax outbreaks and emphasizes the need for a true One Health approach to effectively prevent and control this neglected zoonosis.

Appendix Additional information on spatiotemporal patterns of anthrax, Vietnam, 1990–2015.
