# Supplementary material for: Spatiotemporal Patterns of Anthrax, Vietnam, 1990–2015
Source: Emerg Infect Dis. 2022 Nov;28(11):2206–13. doi: 10.3201/eid2811.212584 (PMC9622238; doi:10.3201/eid2811.212584)
Supplement: Appendix — Additional information on spatiotemporal patterns of anthrax, Vietnam, 1990–2015. [file 21-2584-Techapp-s1.pdf]

Article DOI: https://doi.org/10.3201/eid2811.212584

Spatiotemporal Patterns of Anthrax, Vietnam, 1990–2015

$$\ln\left(\frac{P_t}{P_0}\right)/n$$

Appendix Equation. United Nations average annual rate of change, where n is the length of the time period and P is the population.

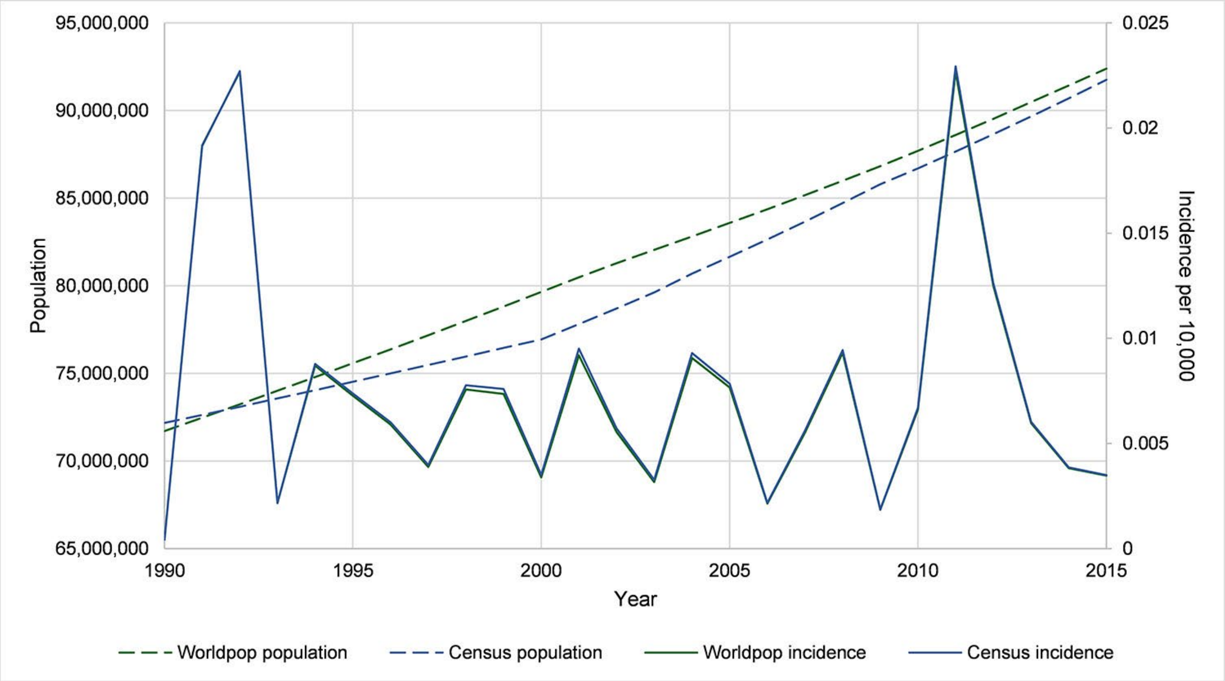

Appendix Figure 1. Comparison between WorldPop population estimates and census-based population estimates in Vietnam. Results indicate that the national human anthrax incidence rates were similar regardless of the population method used.

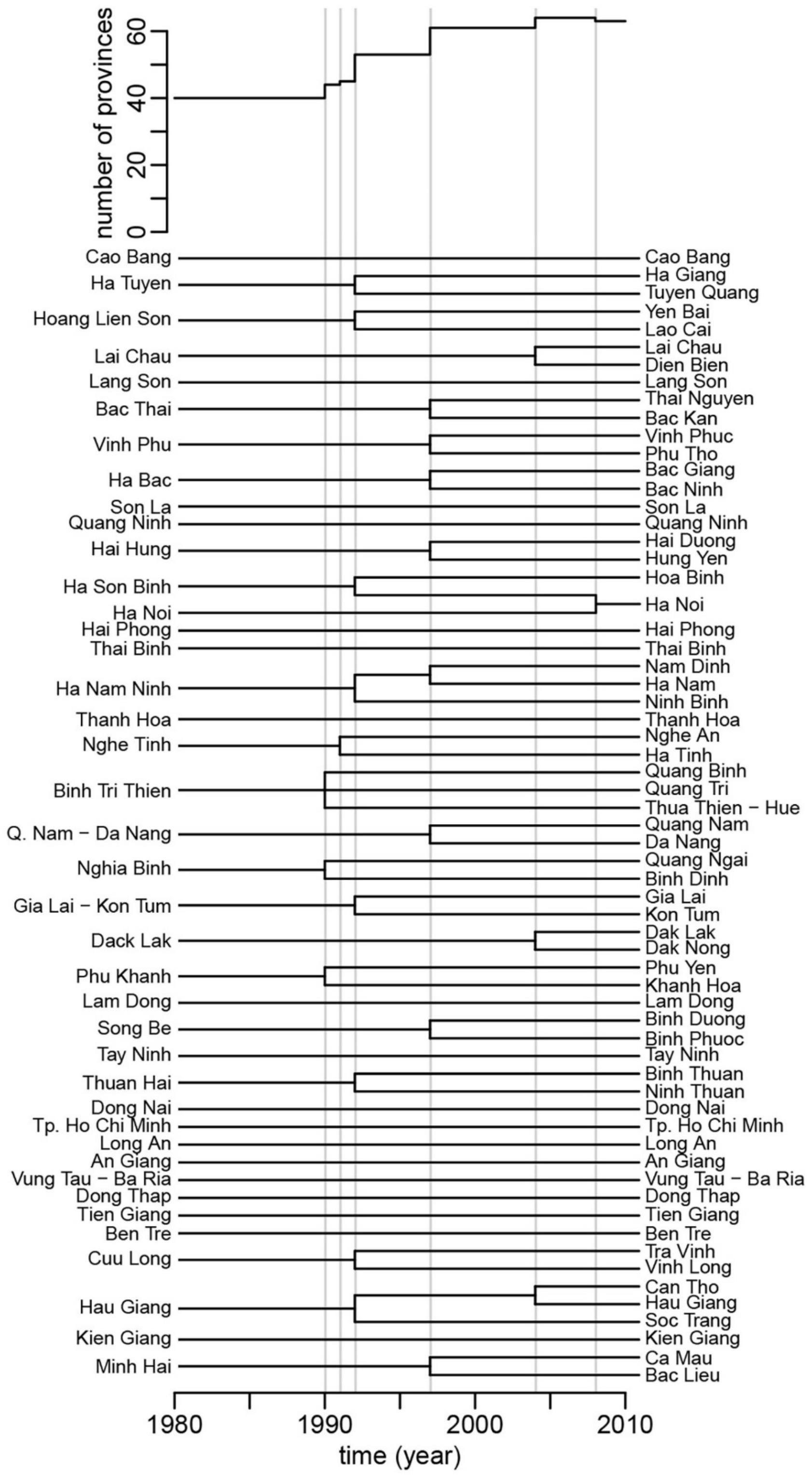

Appendix Figure 2. Province administrative boundary separations and merges for Vietnam during 1980–2010. Years in which the number of total provinces in Vietnam changed are marked by a vertical gray line (*1*).

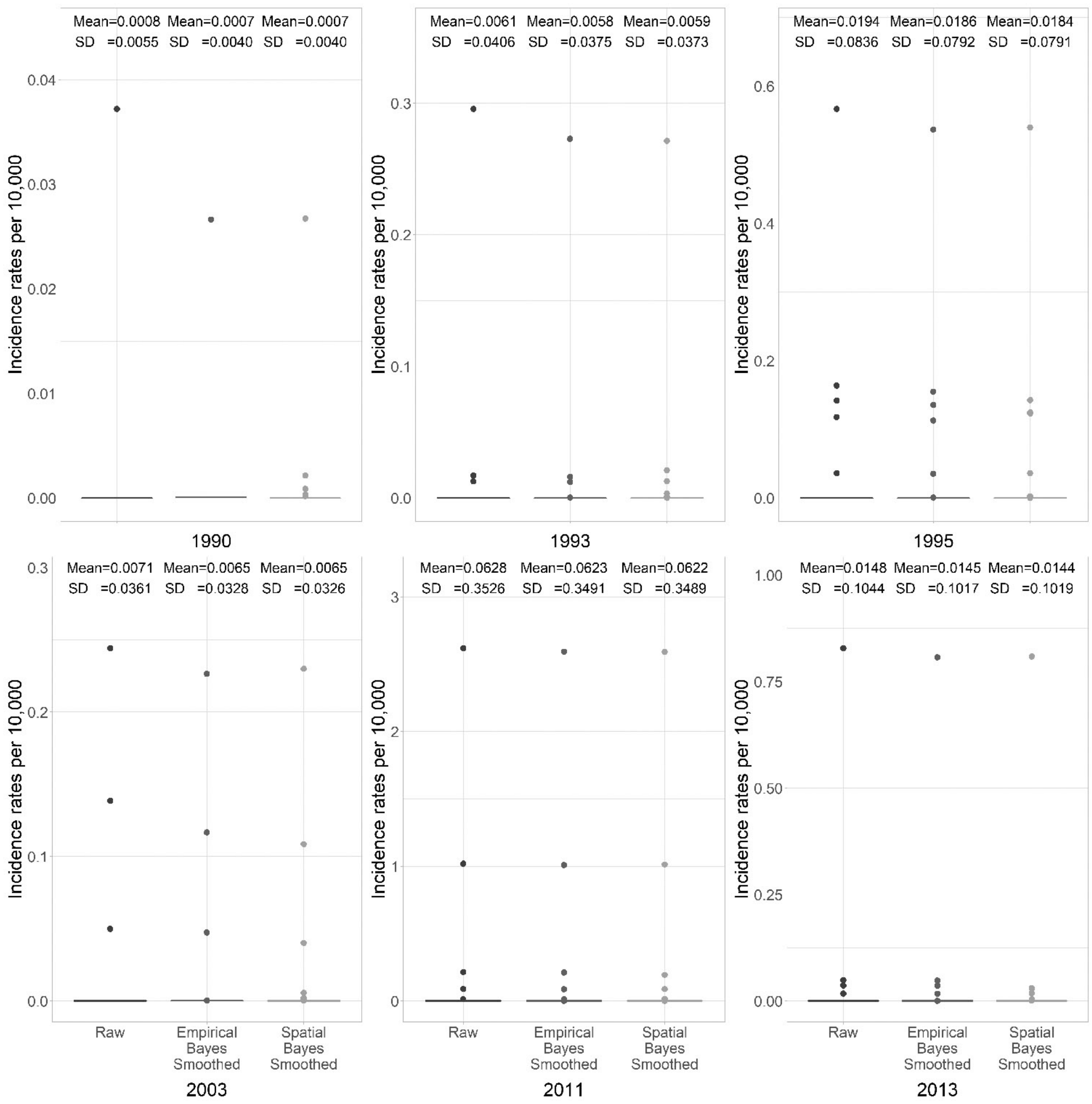

Appendix Figure 3. Box plots comparing raw rates, Empirical Bayes smoothing, and Spatial Bayes smoothing of anthrax incidence rates in Vietnam during 1990, 1993, 1995, 2003, 2011, and 2013.

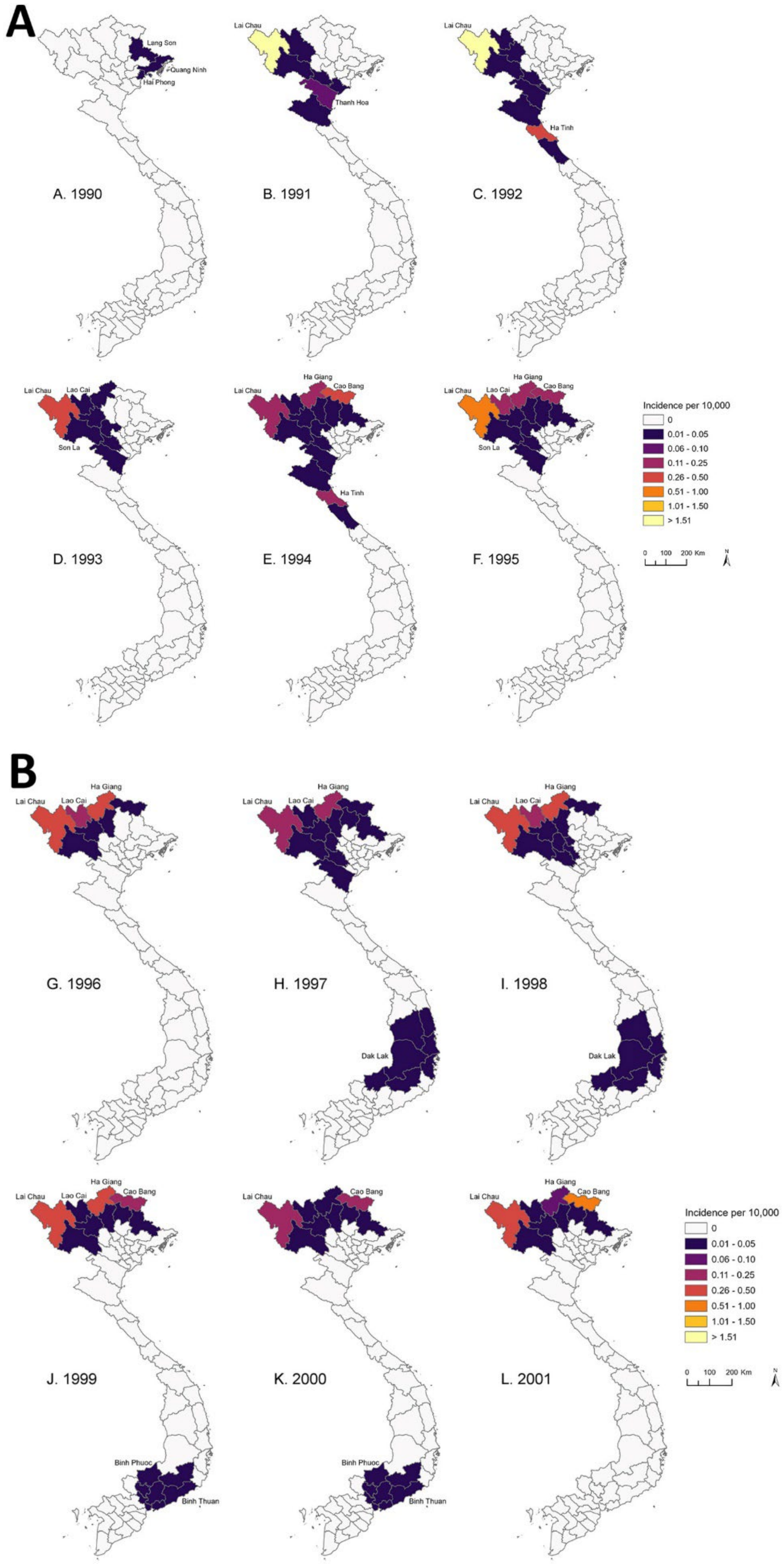
A
Lang Son
Quang Ninh
Hai Phong
A. 1990
Lai Chau
Thanh Hoa
B. 1991
Lai Chau
Ha Tinh
C. 1992
Lai Chau
Lao Cai
Son La
D. 1993
Ha Giang
Lai Chau
Cao Bang
Ha Tinh
E. 1994
Ha Giang
Lai Chau
Lao Cai
Cao Bang
Son La
F. 1995
Incidence per 10,000
0
0.01 - 0.05
0.06 - 0.10
0.11 - 0.25
0.26 - 0.50
0.51 - 1.00
1.01 - 1.50
> 1.51
0 100 200 Km
N
B
Ha Giang
Lai Chau
Lao Cai
G. 1996
Ha Giang
Lai Chau
Lao Cai
Dak Lak
H. 1997
Ha Giang
Lai Chau
Lao Cai
Dak Lak
I. 1998
Ha Giang
Lai Chau
Lao Cai
Cao Bang
Binh Phuoc
Binh Thuan
J. 1999
Lai Chau
Cao Bang
Binh Phuoc
Binh Thuan
K. 2000
Ha Giang
Lai Chau
Cao Bang
L. 2001
Incidence per 10,000
0
0.01 - 0.05
0.06 - 0.10
0.11 - 0.25
0.26 - 0.50
0.51 - 1.00
1.01 - 1.50
> 1.51
0 100 200 Km
N

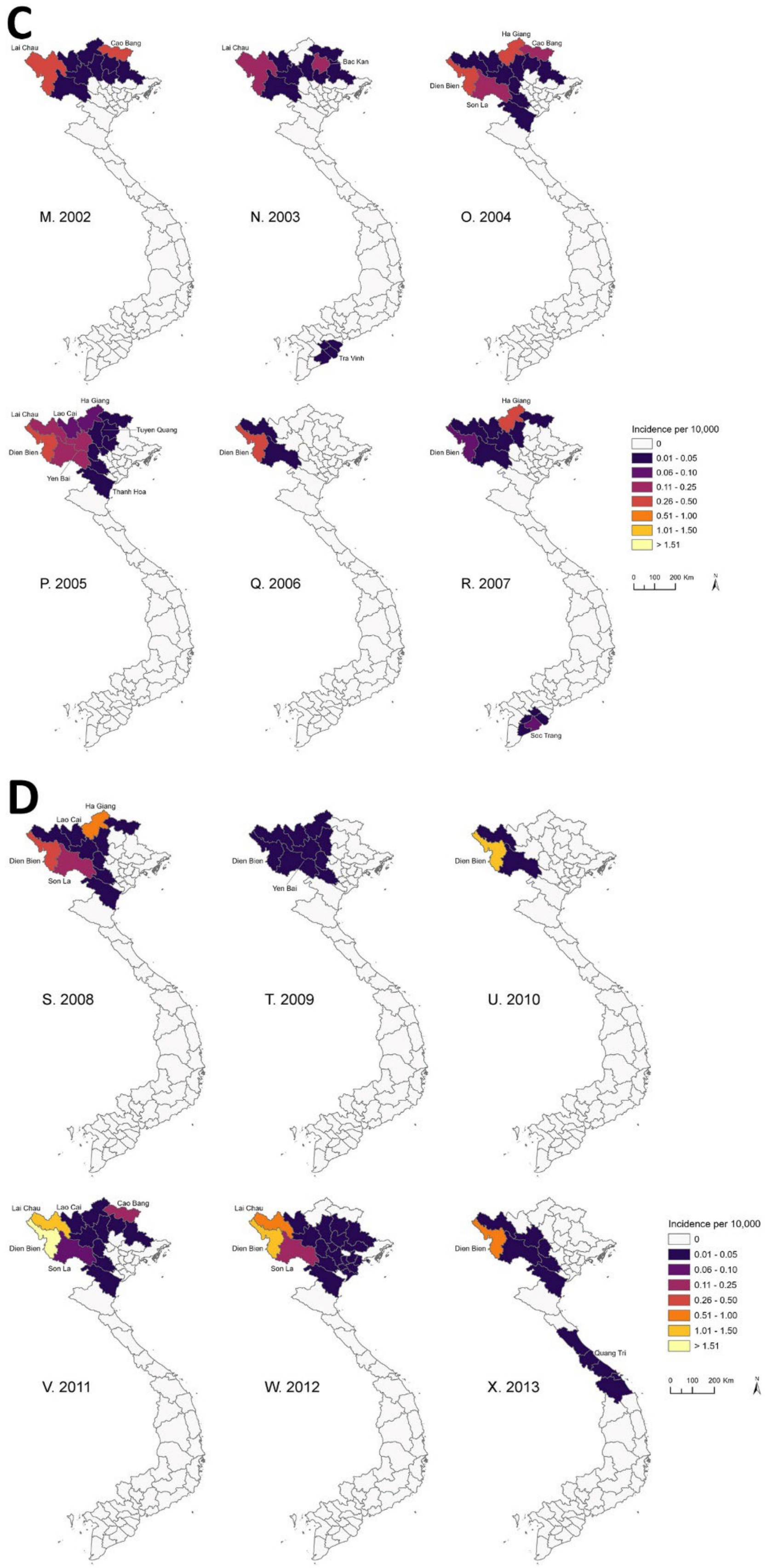
C
Lai Chau
Cao Bang
M. 2002
Lai Chau
Bac Kan
Tra Vinh
N. 2003
Ha Giang
Cao Bang
Dien Bien
Son La
O. 2004
Ha Giang
Lai Chau
Lao Cai
Tuyen Quang
Dien Bien
Yen Bai
Thanh Hoa
P. 2005
Dien Bien
Q. 2006
Ha Giang
Dien Bien
Soc Trang
R. 2007
Incidence per 10,000
0
0.01 - 0.05
0.06 - 0.10
0.11 - 0.25
0.26 - 0.50
0.51 - 1.00
1.01 - 1.50
> 1.51
0 100 200 Km
N
D
Ha Giang
Lao Cai
Dien Bien
Son La
S. 2008
Dien Bien
Yen Bai
T. 2009
Dien Bien
U. 2010
Lai Chau
Lao Cai
Cao Bang
Dien Bien
Son La
V. 2011
Lai Chau
Dien Bien
Son La
W. 2012
Dien Bien
Quang Tri
X. 2013
Incidence per 10,000
0
0.01 - 0.05
0.06 - 0.10
0.11 - 0.25
0.26 - 0.50
0.51 - 1.00
1.01 - 1.50
> 1.51
0 100 200 Km
N

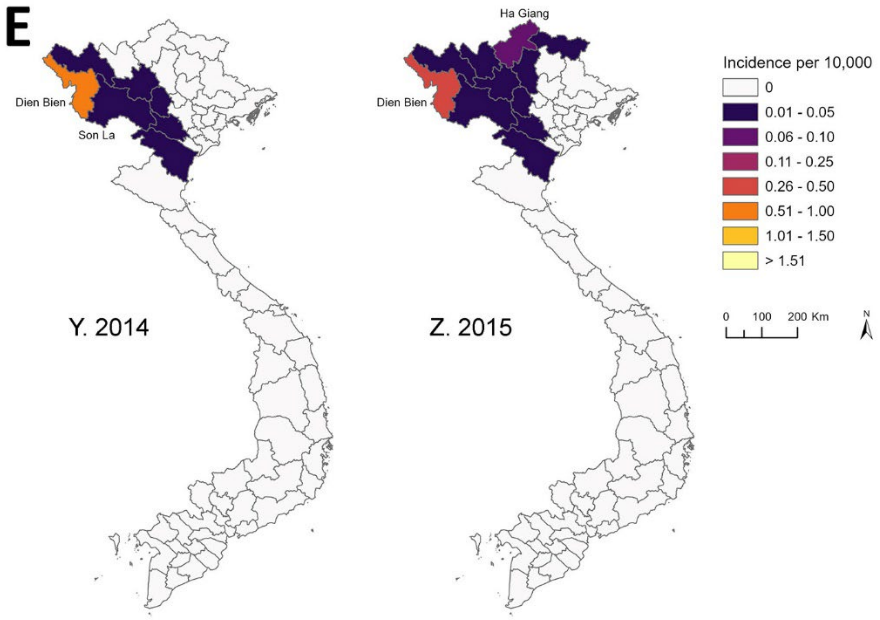

Appendix Figure 4. A–E) Spatial Bayes smoothed human anthrax incidence rates for every year of the study, Vietnam, 1990–2015.

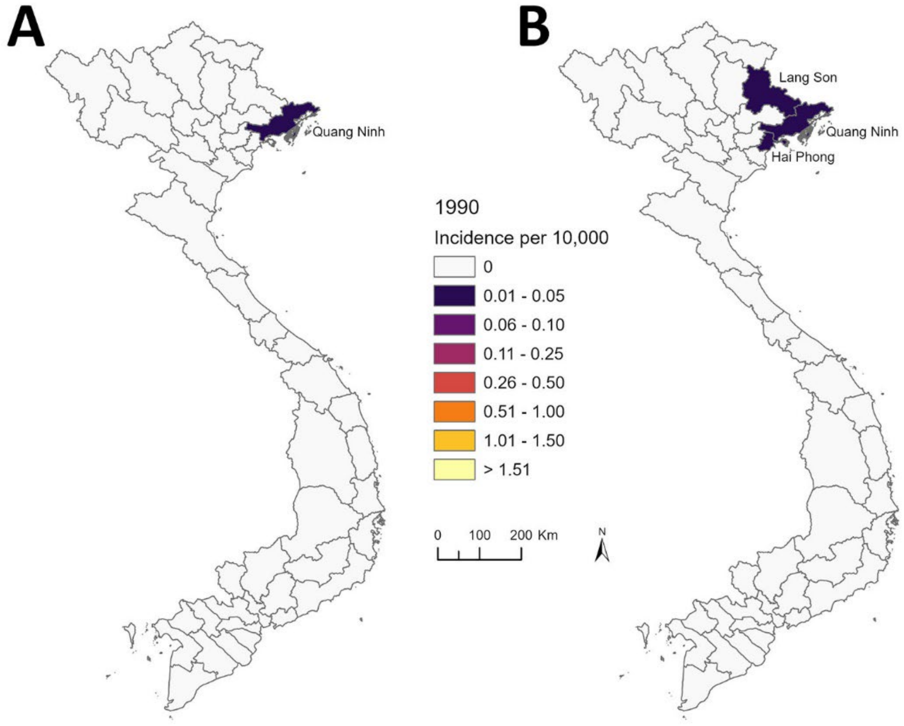

Appendix Figure 5. A GIF of the yearly crude (A) and Spatial Bayes smoothed (B) anthrax incidence rates in provinces of Vietnam from 1990 to 2015. 21-2584-App-F5.gif

Reference

1. Pham QT. The epidemiology and control of human influenza in Vietnam. 2014 [cited 2021 Nov 30]. http://oro.open.ac.uk/id/eprint/39878
